# Age-related accumulation of B-1 cell progenitors in mice reflects changes in miR15a/16-1 expression and radioresistance capacity

**DOI:** 10.1186/s40164-023-00390-6

**Published:** 2023-03-06

**Authors:** Olívia F Souza, Vivian C de Oliveira, Gabriel J. F. Rodrigues, Lucas V. S. Costa, Fernanda Corado, Ana F. Popi

**Affiliations:** Laboratory of Ontogeny of Lymphocytes, Departamento de Microbiologia, Imunologia e Parasitologia, Universidade Federal de São Paulo, Rua Botucatu, 862, 4th floor, São Paulo, 04134090 Brazil

**Keywords:** B-1 cell progenitor, miR15a/16-1, Chronic Lymphocytic Leukemia, Apoptosis, Radioresistance

## Abstract

Hyperproliferative diseases such as Chronic Lymphocytic Leukemia (CLL) and Systemic Lupus Erythematosus (SLE) are potentially related to some disturbance in the apoptosis pathway, specifically in B-1a cells (CD5^+^). Accumulation of B-1a cells in lymphoid organs, bone marrow or periphery is observed in some leukemia experimental murine models along aging. It is known that aging also increases the healthy B-1 cell population. However, it is not yet clear if it happens due to self-renewal of mature cells or proliferation of progenitor cells. Herein we demonstrated that the B-1 cell precursor population (B-1p) from bone marrow of middle-aged mice is higher than from young mice. Also, these aged cells are more resistant to irradiation and have downregulation of microRNA15a/16. Alterations in these microRNAs expression and in *Bcl-2* regulation were already described in human hematological malignancies and new therapeutically approaches focus on that axis. This finding could explain the early events related to cell transformation during aging and correlate with beginning of symptoms in hyperproliferative diseases. Moreover, studies have already reported these pro-B-1 as a contributor to the origin of other leukemia (Acute Myeloid Leukemia - AML). Our results point to a possible relation between B-1 cell precursors and hyperproliferation during aging. We hypothesized that this population could be maintained until the mature status of the cell or reveal changes that result in re-activation of precursor in adult bone marrow, culminating in accumulation of B-1 cells later. Based on this, B-1 cell progenitor could represent an origin for B cell malignancies and a new candidate target to diagnose and treatments in the future.

**Supplementary Information:**

The online version contains supplementary material available at 10.1186/s40164-023-00390-6.

To the editor,

B cells are subdivided in B-1 and B-2 cells, that play different roles in the immune system. Mice B-1 progenitor population (pro-B-1-stage) is described and is distinct from B-2 progenitor [1]. The human B-1p population is yet to be fully characterized but studies showed the development from the same hematopoietic stem cells (HSCs) that give rise to B-2 cells [2].

There is a proposal that some mature human B-cell malignancies and autoimmune diseases could arise from B-1 cells [3, 4]. Accumulation of B-1 cells was seen in New Zealand Black/White mice (NZB/NZW) [5], in patients with SLE [6] and it was described an aged-dependent increase of B-1 cells in NZB/NZW, BALB/c and CBA mice [7]. Other characteristics could be seen in CLL patients and B-1 from NZB/NZW strain, such as presence of the asymptomatic precursor, downregulation of microRNA15a/16-1 and radioresistant capacity to gamma ionization (8Gy) [8, 9]. Despite the association between B-1 cells, CLL and aging, the origin of B-1 cell accumulation is not elucidated. We investigated the role of B-1p cells as a source of B-1 cell malignancies.

We isolated B-1p cells from young (8 weeks old) and middle-aged mice (30 weeks old). First, there is an increase of B-1p cells in middle-aged mice in relation to the young group (p=0.0391), as shown in Fig. 1B. Fig. 1A and C also show increased B-1p in middle-aged as represented by percentage of parent. The absolute number of events also reveals that difference (p=0.0453). Other study showed the accumulation of B-1p cells in older mice (~2 years) [10], however, our aim is to identify changes in B-1p population before that time point.

**Fig. 1:**
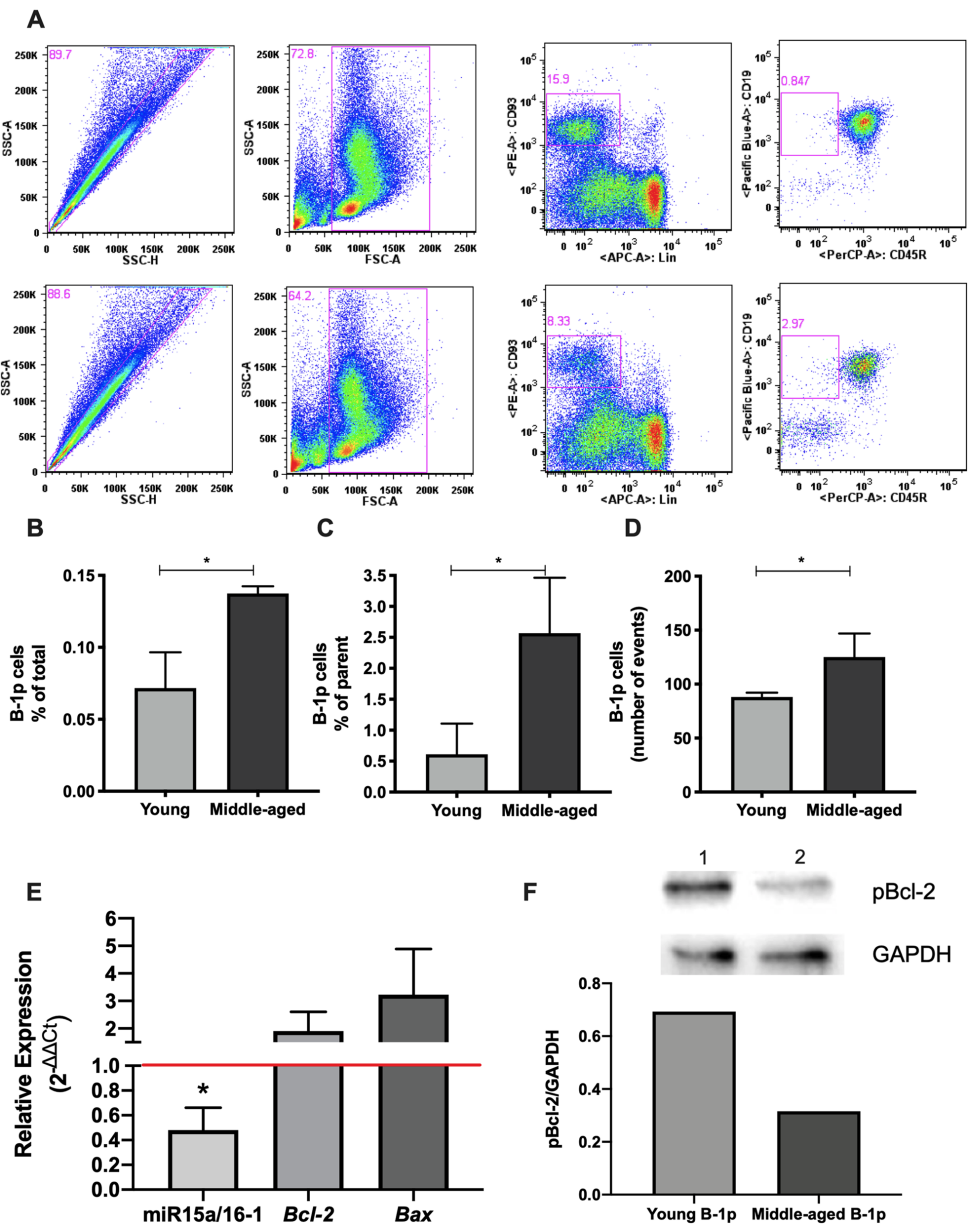
B-1 cell progenitor accumulates in middle-aged C57Bl/6 mice and presents downregulation in miR-15a/16 expression, but no difference in Bcl-2 expression.** A** Gates strategies used to evaluate the percentage of B-1p in bone marrow of young (above) or middle-aged (below) mice. First, the single cells were selected (SSC-A x SSC-H) and the population was defined (SSC-A x FSC-A). After that, Lin^-^CD93^+^ cells were selected followed up to CD45R^-/low^CD19^+^ selection of B-1p phenotype. **B** Frequency of total B-1 progenitor cells (Lin^-^CD45R^-/low^CD19^+^CD93^+^) present in bone marrow of young (0.071% ± 0.0249) and middle-aged mice (0.1375% ± 0.0317). Student’s t test: t(5)=3.512, p=0.0391. **C** Frequency of parent of B-1p cells (Lin^-^CD45R^-/low^CD19^+^CD93^+^) present in bone marrow of young (0.6121% ± 0.4943) and middle-aged (2.5667% ±0.8958) mice. Student’s t test t(5)=3.309, p=0.0297. **D** Absolute number of B-1 progenitor cells (Lin^-^CD45R^-/low^CD19^+^CD93^+^) present in bone marrow of young (88 ± 4) and middle-aged (125 ± 21.9) mice. Student’s t test t(5)=2.875, p=0.0453. Figure. 1A is representative of 3 animals per group evaluated separately. The experiment was repeated with a pool of cells from 10 animals per group. Figures 1B-D represent statistics of three independent experiments combined. **E** These samples were normalized with B-1p samples from young mice and the endogenous gene was *u6* snRNA to miRNA and *ß-2 microglobulin* to apoptotic genes. 2^-ΔΔCt^ method was used. This experiment was performed in triplicate with 4 different biological samples and the average of 4 experiments is shown. Student’s t test t(8)=2.77; p=0.033; cohend’=1.96 (high effect size). **F** Levels of Bcl-2 phosphorylation in young or middle-aged B-1p cells. Lanes 1 and 2 represent young B-1p and middle-aged B-1p, respectively. The graph shows the pBcl-2 and GAPDH relation. This experiment was performed with 2 biological samples (10 animals per group resulting in a pool of cells)

In samples from older animals, miR15a/16-1 is downregulated (p=0.033) whereas *Bcl-2* has tendency to increase (Fig. 1E). The analysis of Bcl-2 phosphorylation in the progenitor revealed that the protein is less phosphorylated in older precursors (Fig. 1F). Since phosphorylation indicate activity, we did not show that Bcl-2 is the responsible for cell accumulation with aging. Bcl-2 gene expression also did not present statistical significance between groups. Considering this, other targets for miR15a/16-1 remains to be investigated. The finding that B-1p cells from middle-aged mice accumulates and already present miR15a/16 downregulation is in accordance with the hypothesis that this could be the time of appearing changes that could culminate in B-1 accumulation in future, in aging and disease. Corroborating this, a study demonstrated that miR-15a deficient B-1p cells repopulated irradiated recipients and produced elevated numbers of B-1 cells [11]. Yet, other investigations about this microRNA, BCL-2 and activity of other apoptosis regulators are necessary to elucidate progenitor accumulation.

When we observed the viability of B-1p *in vitro*, we found more viable cells from middle-aged mice in cell culture after 72h in comparison with young cells (Fig. 2A). Further, these older B-1p cells also presented minor levels of median fluorescence intensity, indicating higher proliferation (Additional file 1: Fig. S1 A-C) and more proliferating cells after 72h in culture (Fig. 2B). So, we conclude that the middle-aged B-1p is more able to survive and proliferate in vitro than B-1 p cells from young mice. Accumulated changes in aging impact survival and proliferation index of cells.

**Fig. 2:**
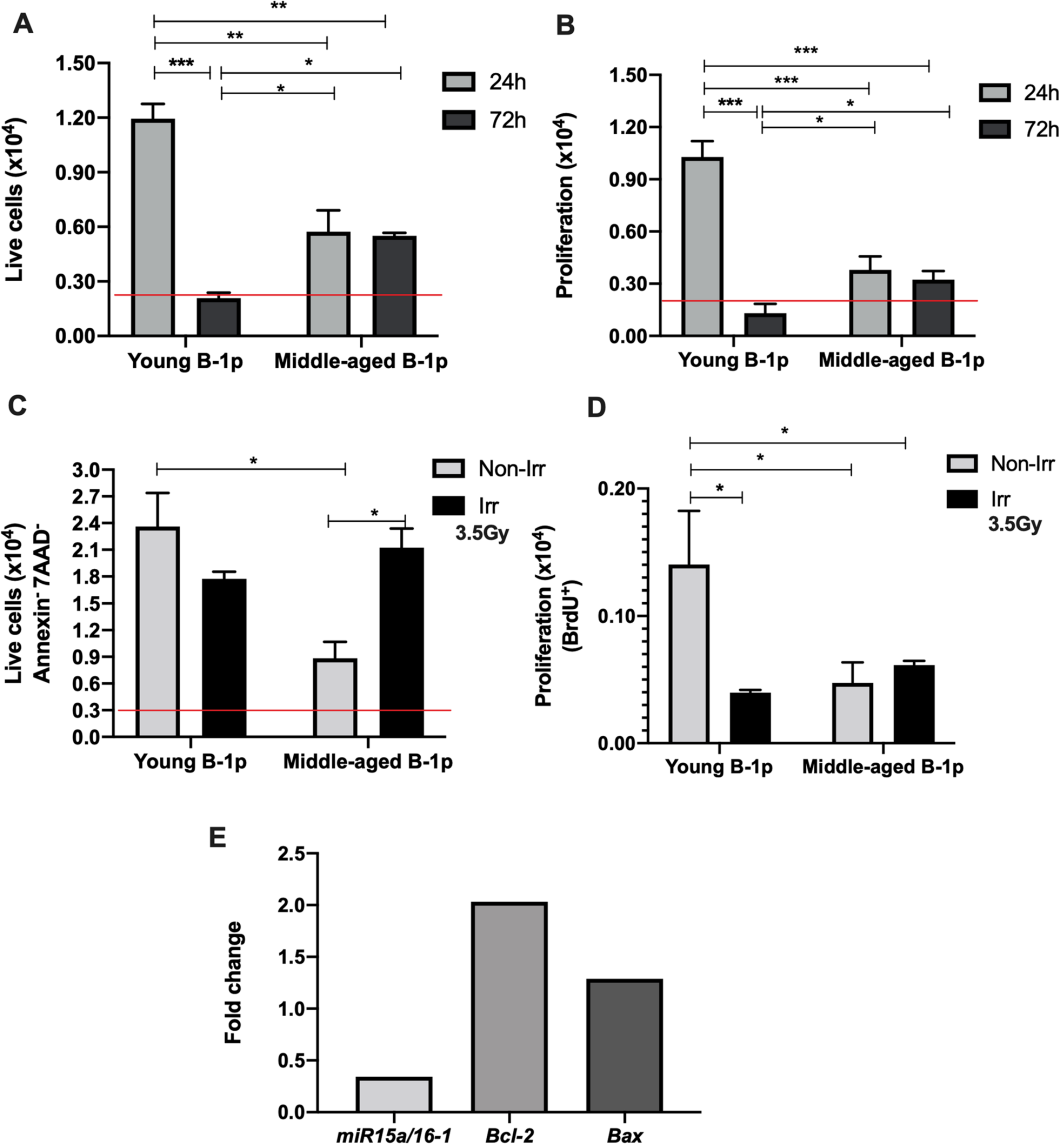
B-1p cells from middle-aged mice survive and proliferate longer in cell culture after 72h and present radioresistance capacity after 3.5Gy of gamma-ionization. **A** Absolute number of live B-1p cells (PI^-^). Two-way ANOVA F(3,8)=13.2 p=0.002. Differences are observed between young 24h (1.030 ± 0.29) and young 72h (0.173 ± 0.064; p<0.001), young 24h and aged 24h (0.570 ± 0.118; p<0.001), young 24h and aged 72h (0.491 ± 0.1043; p=0.002), young 72h and aged 72h (p=0.036) and young 72h with aged 24h (p=0.020). Red line represents the initial number of seeded cells (3x10^3^). **B** Absolute number of live cells in proliferation (levels of median celltrace violet fluoresence intensity). Two-way ANOVA F(3,8)=18.7 p<0.001. Differences are observed between young 24h (1.028 ± 0.99) and young 72h (0.130 ± 0.05; p<0.001), young 24h and aged 24h (0.380 ± 0.07; p<0.001), young 24h and aged 72h (0.323 ± 0.005; p<0.001), young 72h and aged 72h (p=0.038) and young 72h with aged 24h (p=0.011). Red line represents the initial number of seeded cells (3x10^3^). **C** Absolute number of live cells (Annexin^-^7AAD^-^) after 72h in co-culture with OP9 cells. This represents the relation between cell number counted after culture and the percentage of cells annexin^-^7AAD^-^ (Additional file 1: Fig. S2). One-way ANOVA F_welch_(3, 2.42)=20.9; p=0.029. Differences between groups young non-irradiated (2.360 ± 0.3796) and middle-aged non-irradiated (0.884 ± 0.1835; p=0.012) and middle-aged non-irradiated with middle-aged irradiated (2.125 ± 0.2141; p=0.022) are shown. **D** Absolute number of cells that had incorporated BrdU. This represents the relation between cell number counted after culture and the percentage of cells stained with anti-BrdU (Additional file 1: Fig. S3). One-way ANOVA F(3,6)=11.6; p=0.007; Differences between groups young non-irradiated (0.1403 ± 0.04) and middle-aged non-irradiated (0.0474 ± 0.01; p=0.008), young non-irradiated with middle-aged irradiated (0.0615 ± 0.003; p=0.018) and young non-irradiated with young irradiated (0.0398 ± 0.002; p=0.009) are shown. These graphics represent two independent experiments performed in triplicate. Red line represents the initial number of seeded cells (2.5x10^3^). **E** Aged B-1p irradiated or aged non-irradiated samples were normalized with the respective sample of young mice and endogenous gene were *ß2 microglobulin* for *Bcl-2* and *Bax* analysis or *U6* for miR15a/16. Relative expression was evaluated by 2^-ΔΔCt^ and here contains the ratio (fold change) between irradiated and non-irradiated group after 2^-ΔΔCt^ calculus in each group (young or middle-aged). This graphic represents 2 experiments performed in triplicates

Previous data from our group reported that a small percentage of B-1 cells are able to survive *in vitro* after 3.5Gy dose of irradiation and acquired characteristics of neoplastic cells (B-CLL like cells) [12]. We found a decrease in live cell numbers in cell culture with young irradiated B-1p cells, in comparison with non-irradiated control group. However, when B-1p cells from middle-aged mice were irradiated, these cells survived and an increase in the number of cells is observed in comparison to control group (Fig. 2C). The irradiation also influences proliferation, as demonstrated in Fig. 2D. Young cells do not proliferate after irradiation but old cells augment proliferation after this process (data are presented at Fig. 2D and in Additional file 1: Fig. S3). Based on the fold change analysis between irradiated and non-irradiated group, it is possible to observe that middle-aged irradiated cells have higher expression of anti-apoptotic gene *Bcl-*2 (Fig. 2E). The *Bax* gene did not change between these groups. Moreover, older irradiated cells demonstrated a downregulation of miR15a/16-1 (consistent with levels of *Bcl-2*). However, a threshold may need to be attained to reveal the role of Bcl-2. These data could explain that B-1 precursors obtained from middle-aged animals survive to irradiation *in vitro* and also have higher proliferation index.

These findings demonstrated that B-1p have molecular changes induced by aging that are augmented with radiation and could lead progenitors to acquire malignant potential.

## Supplementary Information

**Additional file 1: Figure S1.** B-1p cells from middle-aged mice proliferate in vitro more than young B-1p cells. **A)** Celltrace fluorescence histogram of young (blue) or middle-aged (green) B-1p after 24h in cell culture. Purple bar indicates cells stained with celltrace in t0. **B)** Celltrace fluorescence histogram of young (blue) or middle-aged (green) B-1p after 72h in cell culture. Purple bar indicates cells stained with celltrace in t0. **C)** Median fluorescence intensity of celltrace reagent in proliferating progenitors. Two-way ANOVA F(3,8)=6.32 p=0.017. Differences are observed between aged 24h (6766 ± 548) and all other groups: young 24h (2302 ± 490; p<0.001), young 72h (3757 ± 147; p<0.001) and aged 72h (2734 ± 195; p<0.001) and young 24h with young 72h (p=0.023). This figure represents two different experiments performed in triplicate. **Figure S2.** B-1 progenitor cells from young and middle-aged mice survives in vitro after irradiation. **A)** Gates strategies revealing live B-1p cells (Annexin^-^7AAD^-^), from young (above) and middle-aged (below) mice. **B)** Positive control of cell death. B-1p cells from this same experiment were collected after cell sorting and placed on dry-bath at 95º for 5 minutes. Cells were stained with Annexin V and 7AAD as previously described. **C)** Percentage of live cells (Annexin^-^7AAD^-^) found after irradiation and cell culture. **Figure S3.** B-1 progenitor cells from young and middle-aged mice survives in vitro after irradiation but differs in proliferation rates. **A) **Gates strategies revealing B-1p cells, from young (above) and middle-aged (below) mice, that incorporated BrdU marker. **B) **Percentage of cells that incorporated BrdU marker after irradiation and cell culture period. Two-way ANOVA F(3,5)=6.03; p=0.041. Difference between young ctrl non irr (1.63 ± 0.375) and young irr (4.1 ± 0.955; p=0.036) is shown.

## Data Availability

The data that support the findings of this study are available from the corresponding author upon reasonable request.

## Abbreviations

CLL: Chronic Lymphocytic Leukemia
SLE: Systemic Lupus Erythematosus
B-1p: B-1 cell progenitor
Bcl-2: B cell lymphoma 2
HSCs: Hematopoietic Stem Cells
NZB/NZW: New Zealand Black/White mice
SLE: Systemic Lupus Erythematosus
