# Supplementary material for: Age-related accumulation of B-1 cell progenitors in mice reflects changes in miR15a/16-1 expression and radioresistance capacity
Source: Exp Hematol Oncol. 2023 Mar 6;12:24. doi: 10.1186/s40164-023-00390-6 (PMC9987129; doi:10.1186/s40164-023-00390-6)
Supplement: Supplementary file 1 — Additional file 1: Figure S1. B-1p cells from middle-aged mice proliferate in vitro more than young B-1p cells. A) Celltrace fluorescence histogram of young (blue) or middle-aged (green) B-1p after 24h in cell culture. Purple bar indicates cells stained with celltrace in t0. B) Celltrace fluorescence histogram of young (blue) or middle-aged (green) B-1p after 72h in cell culture. Purple bar indicates cells stained with celltrace in t0. C) Median fluorescence intensity of celltrace reagent in proliferating progenitors. Two-way ANOVA F(3,8)=6.32 p=0.017. Differences are observed between aged 24h (6766 ± 548) and all other groups: young 24h (2302 ± 490; p<0.001), young 72h (3757 ± 147; p<0.001) and aged 72h (2734 ± 195; p<0.001) and young 24h with young 72h (p=0.023). This figure represents two different experiments performed in triplicate. Figure S2. B-1 progenitor cells from young and middle-aged mice survives in vitro after irradiation. A) Gates strategies revealing live B-1p cells (Annexin-7AAD-), from young (above) and middle-aged (below) mice. B) Positive control of cell death. B-1p cells from this same experiment were collected after cell sorting and placed on dry-bath at 95º for 5 minutes. Cells were stained with Annexin V and 7AAD as previously described. C) Percentage of live cells (Annexin-7AAD-) found after irradiation and cell culture. Figure S3. B-1 progenitor cells from young and middle-aged mice survives in vitro after irradiation but differs in proliferation rates. A) Gates strategies revealing B-1p cells, from young (above) and middle-aged (below) mice, that incorporated BrdU marker. B) Percentage of cells that incorporated BrdU marker after irradiation and cell culture period. Two-way ANOVA F(3,5)=6.03; p=0.041. Difference between young ctrl non irr (1.63 ± 0.375) and young irr (4.1 ± 0.955; p=0.036) is shown. [file 40164_2023_390_MOESM1_ESM.pdf]

# *Supplementary Material*

## 1. Material and Methods

### 1.1 Mice

C57BL/6 female mice, 8-10 weeks (young) or 30 weeks (middle-aged), were obtained from the Centro de Desenvolvimento de Modelos Experimentais para Medicina e Biologia (CEDEME) of the Universidade Federal de São Paulo (UNIFESP). All the animals were maintained under pathogen free conditions. All procedures described here were approved by the Ethical Committee from UNIFESP (2017/3576280617, 2018/7292050618 and 2019/1374110219).

### 1.2 Quantification of B-1 cell precursor (B-1p) in young and older animals

Cells from the bone marrow washout from mice were collected in RPMI 1640 medium and were isolated from both femurs and tibias of each animal. Three animals per group were evaluated separately. In the next experiments, we also evaluated the quantity of B-1 cell progenitors in the bone marrow of young or aged animals, analyzing samples containing a pool of cells from 10 animals per group. Cells were counted and incubated with anti-CD16/CD32 mAb (BD-Pharmingen, California; USA) to block Fcγ RIII/II receptors and stained on ice for 30 min with the monoclonal antibodies against the following molecules: CD3e, CD11c, F4/80, Gr-1, Nk1.1 – conjugated with allophycocyanin (APC), CD93/AA4.1 conjugated with phycoerythrin (PE), CD45R/B220 conjugated with PECy-7 and CD19 conjugated with Pacific Blue. All these antibodies were obtained from BD Biosciences (California, USA). The phenotype of B-1 cell progenitor is defined as: $Lin^{-}CD45R/B220^{-}CD93/AA4.1^{+}CD19^{+}$. Cells were acquired using the BD FACSCanto™II flow cytometer and data were analyzed with FlowJo software v.9.5 (Becton-Dickinson, USA).

### 1.3 Enrichment of B-1 and B-1p cells from young and older animals

B-1 cell population was cell sorted by BD FACSAria II from peritoneal cavity washout (using RPMI1640 medium). Cells were counted and incubated with anti-CD16/CD32 mAb (BD-Pharmingen, California; USA) to block Fcγ RIII/II receptors and stained on ice for 20 min with the monoclonal antibodies against the following molecules: CD3e, CD11c, F4/80, Gr-1, NK1.1 – conjugated with APC (allophycocyanin) and FITC-conjugated monoclonal antibody anti-CD23. All these antibodies were obtained from BD Biosciences (California, USA). We used the negative strategy to purify the B-1 cell population: from the lymphocyte gate (FSC x SSC), a double negative population ($Lin^-CD23^-$) was sorted. After that, an aliquot of sorted cells was stained with CD19 and CD23 to confirm the B-1 cell purity. In all experiments, purity ≥ 95% after cell sorting was considered to perform the following analysis.

Bone marrow was processed and stained as described above (item 2) and B-1p cells were enriched by cell sorting (BD FACSAria II), using the following strategy: from lineage negative population (CD3e, CD11c, F4/80, Gr-1, NK1.1), a negative population to CD45R was selected and from this population, a double positive population to CD19 and CD93 was sorted. Enrichment ≥ 95% was considered to perform the experiments. B-1 cell and B-1 cell precursor enriched populations were obtained from pooled sorting cells from 10-12 mice of each group (young or middle-aged).

**1.4 Proliferation and viability of B-1 or B-1p cells from young and middle-aged mice *in vitro***

B-1 or B-1p cells from middle-aged and young mice were collected and enriched as mentioned before (items 2 and 3) and stained with Celltrace Violet Proliferation kit (Thermo Fisher Scientific, Eugene, OR, USA). $2x10^5$ B-1 cells/well were added in the 96 wells plate or $2.5x10^3$ of B-1p cells/well were added in RPMI with 10% fetal bovine serum (FBS), 1% penicillin and 1% L-glutamine. All these cells were cultivated in triplicate. In B-1 progenitor culture, OP9 cells (ATCC CRL-2749) were seeded 24 hours before the experiment in α−MEM medium + 20% (FBS). These

cells were maintained for 24 and 72h at 37ºC and 5% of $CO_2$. After this period, the supernatant was collected, counted and cells stained with propidium iodide (PI - Thermo Fisher Scientific – Molecular Probes, Eugene, OR, USA) to identify of live and dead cells. These cells were acquired in the FACSCanto II flow cytometer and the results were analyzed in FlowJo 9.5 software. The absolute number of live or in proliferation cells was calculated by the relation between the counted cells (Neubauer Chamber) and the percentage found in the FlowJo analysis. The fluorescence decay with Celltrace reagent was used to calculate MFI and demonstrate proliferation.

**1.5 Analysis of cell viability after irradiation**

After the cell sorting, B-1p cells obtained from young or middle-aged mice were divided in two groups: non-irradiated (control) or irradiated, which was exposed to 3.5 Gy of gamma radiation (Gammacel ®3000 – Nordion International Inc., Ottawa; Canada). B-1p cells were maintained in culture with OP9 cells (ATCC CRL-2749) for 72h. $1x10^4$/well OP9 cells were seeded with α−MEM medium + 20% fetal bovine serum (FBS) 24h before the irradiation experiment, to adhere to plate surface (96 well plate). To exclude OP9 cells from analysis, these cells were previously stained with Celltrace Violet Proliferation Kit (Thermo Fisher Scientific, Eugene, OR, USA). 100μL of RPMI medium + 10% FBS containing $2x10^3$ B-1p was added per well, after discarded 100μL of MEM medium from each well. These co-cultures were incubated at 37ºC with 5% of $CO_2$.

After the incubation period, supernatant was collected, cells counted and cell viability was measured. For this, B-1p cells were stained with PE Annexin V Apoptosis Detection Kit (BD Biosciences), following the manufacturer's protocol. The cells were acquired in FACSCanto II cytometer (BD-Pharmingen, California; USA) and apoptosis was analyzed by FlowJo v9.5 software (Becton-Dickinson, USA). The absolute number calculus was based on the percentage of live cells

(Anexin$^-$7AAD$^-$) found in the flow cytometry analysis and the number of cells detected after culture (counted by Neubauer Chamber).

**1.6 Proliferation analysis of B-1p cells after irradiation**

Irradiated or non-irradiated B-1p cells were also evaluated by BrdU incorporation. Irradiation and culture of cells was performed as described early. BrdU reagent from Apoptosis, DNA damage and Cell Proliferation kit (BD Biosciences) was used immediately after irradiation. $2x10^3$ cells were added, per well, on the OP9 layer in 96 well plates. After 72h in culture, supernatant of each group was collected, counted, cells were treated with DNase to expose BrdU and stained with PerCP-Cy™5.5 Mouse Anti-BrdU antibody. The acquisition was performed at FACSCanto II Flow Cytometer and analyzed by FlowJo 9.5 software. The absolute number calculus was based on the percentage of Brdu$^+$ cells found in the flow cytometry analysis and the number of cells detected after culture (counted by Neubauer Chamber).

**1.7 Gene expression analysis**

After 72h in cell culture post-irradiation, we performed RNA extraction using TRIzol reagent (Thermo Fisher Scientific; Baltics, Lithuania) in young and older B-1p cells, irradiated or not. The reverse transcription was performed with SuperScript IV First-Strand Synthesis (Thermo Fisher Scientific, Baltics, Lithuania) to run a quantitative Polymerase Chain Reaction and evaluate expression of apoptotic genes (*Bcl-2*: CTGCACCTGACGCCCTTCACC/CACATGACCCCACCGAACTCAAAGA and *Bax*: TGAAGACAGGGGCCTTTTTG/AATTCGCCGGAGACACTCG) and miR15a/16-*1* (Taqman probes – Thermo Fisher Scientific). This was analyzed in 7500 software, considering Ct values and

$2^{-\Delta\Delta Ct}$ formula. We perform two different reactions and each of them was carried out in triplicate using one biological sample.

We also evaluated the relative expression of these anti-apoptotic genes and miR15a/16-1 in B-1p cells from young or middle-aged mice in a separated experiment, with collected cells from sorting. Each reaction was carried out in triplicate using at least four biological samples and for the miRNA extraction the miRNeasy mini kit (Qiagen) was used.

qPCRs were performed in Real-Time 7500 Fast (Applied Biosystems), using FAST Sybr Green reagent (Qiagen, Hilden, Germany) to evaluate apoptotic genes or FAST master mix TaqMan reagent (Applied Biosystems, Lithuania) to evaluate miR15a/16-1. The following oligos TaqMan™ were used to specific amplification of miR15a/16-1 (hsa-miR-16 – assay ID 000391) and *U6* (*U6* snRNA – assay ID 001973 - Thermo Fisher Scientific, Foster City, CA, USA). B-1p samples obtained from young mice were used as normalizer (control group) and to calculate the relative expression, we used *B2-microglobulin* (ATGGCTCGCTCGGTGACCCT/TTCTCCGGTGGGTGGCGTGA) or *U6* (*U6* snRNA – assay ID 001973) as endogenous genes. The statistical analysis was made comparing dCt values of target and endogenous genes with Student's t test or ANOVA One-Way, considering 5% of significance.

**1.8 Western-blotting**

Total protein extract from B-1 cell progenitor obtained in young or middle-aged mice and 16ug was applied in a 10% polyacrylamide gel and after electrophoresis, it was transferred to a nitrocellulose membrane. The membrane was blocked with a TBS solution containing 0.1% Tween and 5% blotting-grade blocker (BIORAD - Hercules, CA, USA) and incubated with primary antibodies: pBcl-2 (1:500; Cell Signaling, Danvers, MA, USA) and GAPDH (1:3000; Cell Signaling, Danvers, MA, USA). After incubation with secondary antibody anti-IgG (1:1000 Sigma- St Louis,

MO, USA), the chemiluminescence signal was visualized with Supersignal West Pico Chemiluminescent Substrate (Thermo Fisher Scientific, Eugene, OR, USA) and the UVITEC Cambridge equipment. Images were captured with UVITEC Cambridge software and band intensities were calculated in ImageJ software (NIH).

**1.9 Statistical analysis**

Student's T test was used when comparing two samples, with 5% of significance. One-way ANOVA was used to measure statistics between two or more groups of samples with 5% significance and Tukey was used as a post-hoc test. Requirements of normality and homogeneity were verified before running the tests. Non-homogenic samples were evaluated with Welch correction. All statistical analysis was performed in Jamovi v1.2.0.5 software and $P<0.05$ was considered significant. This significance is represented in figures as: ***, $P < 0.001$; **, $P < 0.01$; *, $P < 0.05$ and n.s., not significant. Results are indicated with Mean ± DP and p value. The graphs were constructed in Graph Pad Prism v.7.

## 2. Supplementary Figures

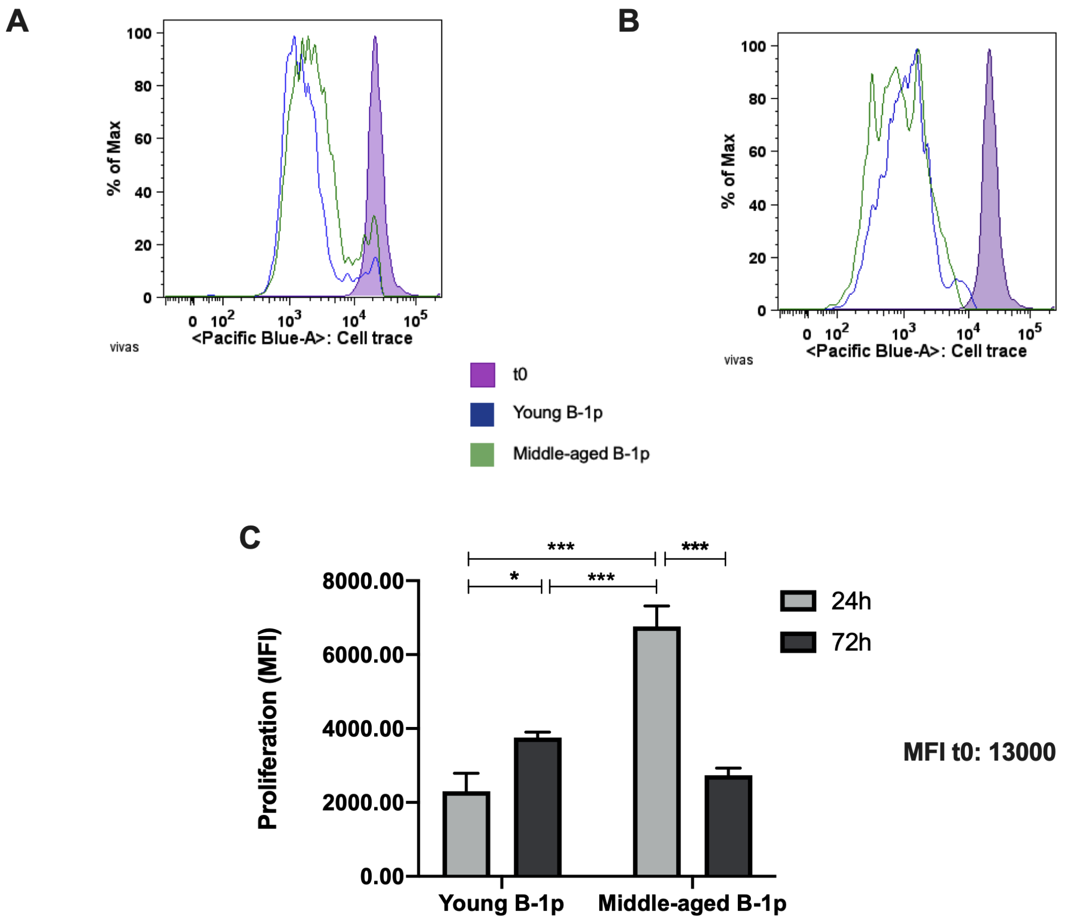


**Supplementary Figure 1: B-1p cells from middle-aged mice proliferate *in vitro* more than young B-1p cells. A)** Celltrace fluorescence histogram of young (blue) or middle-aged (green) B-1p after 24h in cell culture. Purple bar indicates cells stained with celltrace in t0. **B)** Celltrace fluorescence histogram of young (blue) or middle-aged (green) B-1p after 72h in cell culture. Purple bar indicates cells stained with celltrace in t0. **C)** Median fluorescence intensity of celltrace reagent in proliferating progenitors. Two-way ANOVA $F(3,8)=6.32$ $p=0.017$. Differences are observed between aged 24h (6766 ± 548) and all other groups: young 24h (2302 ± 490; $p<0.001$), young 72h (3757 ± 147; $p<0.001$) and aged 72h (2734 ± 195; $p<0.001$) and young 24h with young 72h ($p=0.023$). This figure represents two different experiments performed in triplicate.

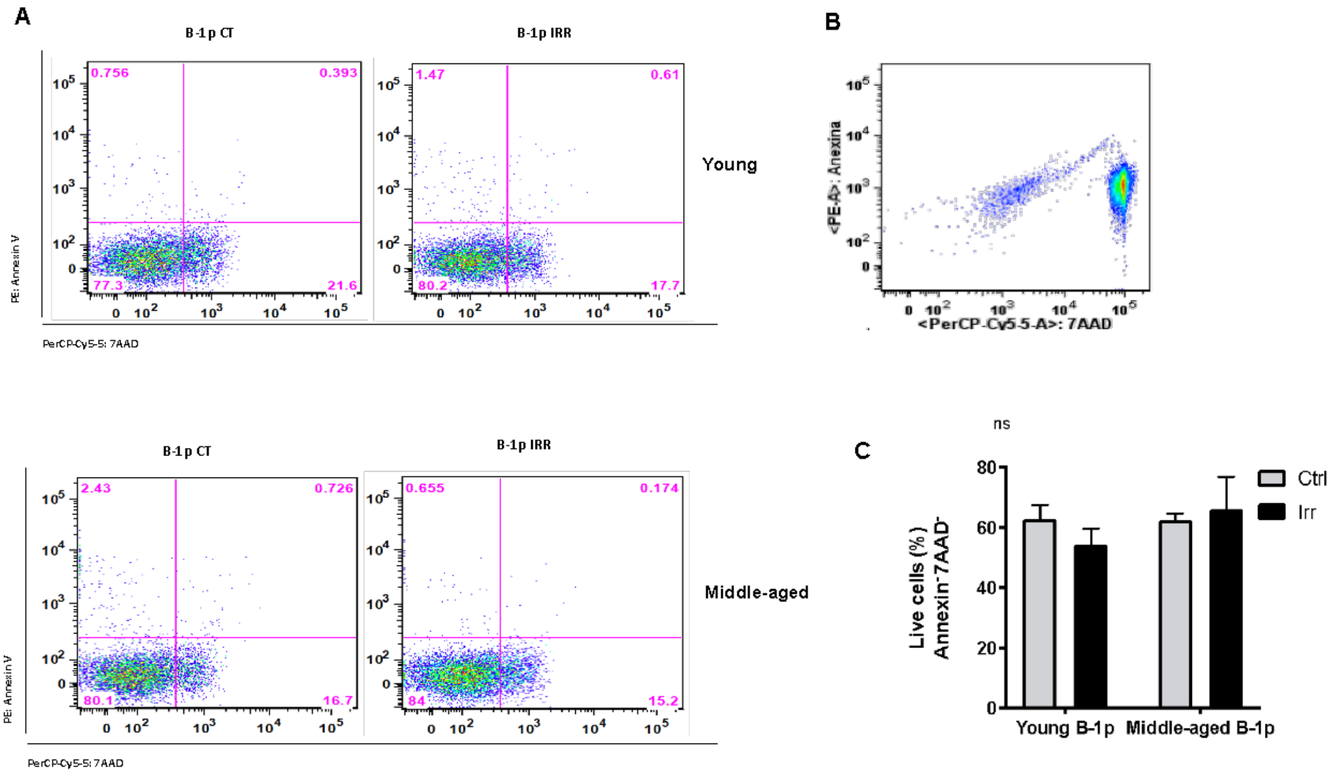


**Supplementary Figure 2: B-1 progenitor cells from young and middle-aged mice survives *in vitro* after irradiation. A** – Gates strategies revealing live B-1p cells (Annexin$^-$7AAD$^-$), from young (above) and middle-aged (bellow) mice. **B** – Positive control of cell death. B-1p cells from this same experiment were collected after cell sorting and placed on dry-bath at 95º for 5 minutes. Cells were stained with Annexin V and 7AAD as previously described. **C** – Percentage of live cells (Annexin$^-$7AAD$^-$) found after irradiation and cell culture.

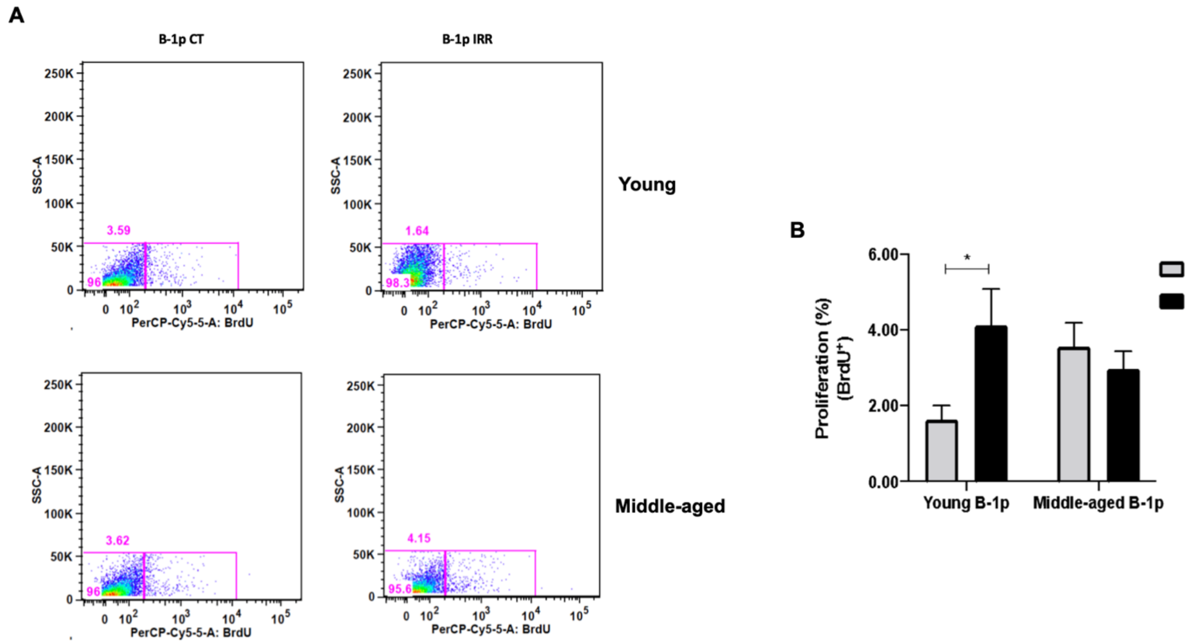


**Supplementary Figure 3: B-1 progenitor cells from young and middle-aged mice survives *in vitro* after irradiation but differs in proliferation rates. A** – Gates strategies revealing B-1p cells, from young (above) and middle-aged (bellow) mice, that incorporated BrdU marker. **B** – Percentage of cells that incorporated BrdU marker after irradiation and cell culture period. Two-way ANOVA $F(3,5)=6{,}03$; $p=0{,}041$. Difference between young ctrl ($1{,}63 \pm 0{,}375$) and young irr ($4{,}1 \pm 0{,}955$; $p=0{,}036$) is shown.
